# Acute Pancreatitis Caused by Fishbone Impaction in the Pancreas: A Case Report

**DOI:** 10.7759/cureus.89327

**Published:** 2025-08-04

**Authors:** Dongyun Hang, Kai Ding, Jie Tang, Beifang Ning, Lingmei Feng

**Affiliations:** 1 Gastroenterology, Shanghai Pudong New Area People's Hospital, Shanghai, CHN; 2 Gastroenterology, The Second Affiliated Hospital of Naval Medical University, Shanghai, CHN

**Keywords:** acute pancreatitis, case report, early diagnosis, foreign body, gastric perforation

## Abstract

Acute pancreatitis (AP) is a common inflammatory disease of the pancreas, which can range from mild to severe and life-threatening. The leading causes of AP include gallstones, alcohol abuse, hypertriglyceridemia, trauma, and pancreatic tumors. Here, we report a rare case of AP caused by the penetration of a fishbone into the pancreas. The treatment of pancreatitis caused by foreign bodies generally requires surgical or endoscopic removal of foreign bodies; otherwise, it may cause severe complications, such as an abscess. However, a repeated CT after transfer found that the foreign body had migrated to the intestinal lumen spontaneously. Therefore, conducting a rigorous pre-intervention evaluation is critical for clinical decision-making. This case underscores the critical roles of early imaging, rapid diagnosis, inter-hospital or multidisciplinary cooperation, and prompt appropriate treatment in managing AP caused by a foreign body.

## Introduction

Acute pancreatitis (AP) is characterized by local and systemic inflammation with a variable clinical course [1,2]. Patients typically present with sudden-onset upper abdominal pain and elevated serum pancreatic enzymes such as amylase and lipase [3]. While most cases are mild, approximately 20% progress to moderate or severe AP with a poorer prognosis [4]. Established etiologies include gallstones (most common) and excessive alcohol consumption; other causes encompass hypertriglyceridemia, medications, trauma, and pancreatic tumors [1,2].

AP secondary to foreign body penetration, particularly by fishbones, is exceptionally rare, with its incidence poorly characterized [4,5]. Although foreign body ingestion is relatively common, most objects pass uneventfully through the gastrointestinal tract [6]. Sharp objects like fishbones typically cause complications in the upper GI tract (e.g., esophageal or gastric). The posterior gastric antrum lies in direct apposition to the pancreatic head and neck, separated only by fused peritoneal layers. Antral peristalsis can mechanically drive impacted sharp foreign bodies into the pancreatic parenchyma, where penetrating trauma and subsequent biochemical cascades trigger pancreatitis [7]. Reports of fish bone-induced pancreatitis are rare, and in the cases, the fish bones were surgically removed [8,9]. However, in the case we report, pancreatitis was caused by a fishbone that unexpectedly migrated into the intestinal lumen and presented spontaneous resolution without any specific intervention.

## Case presentation

**Figure 1:**
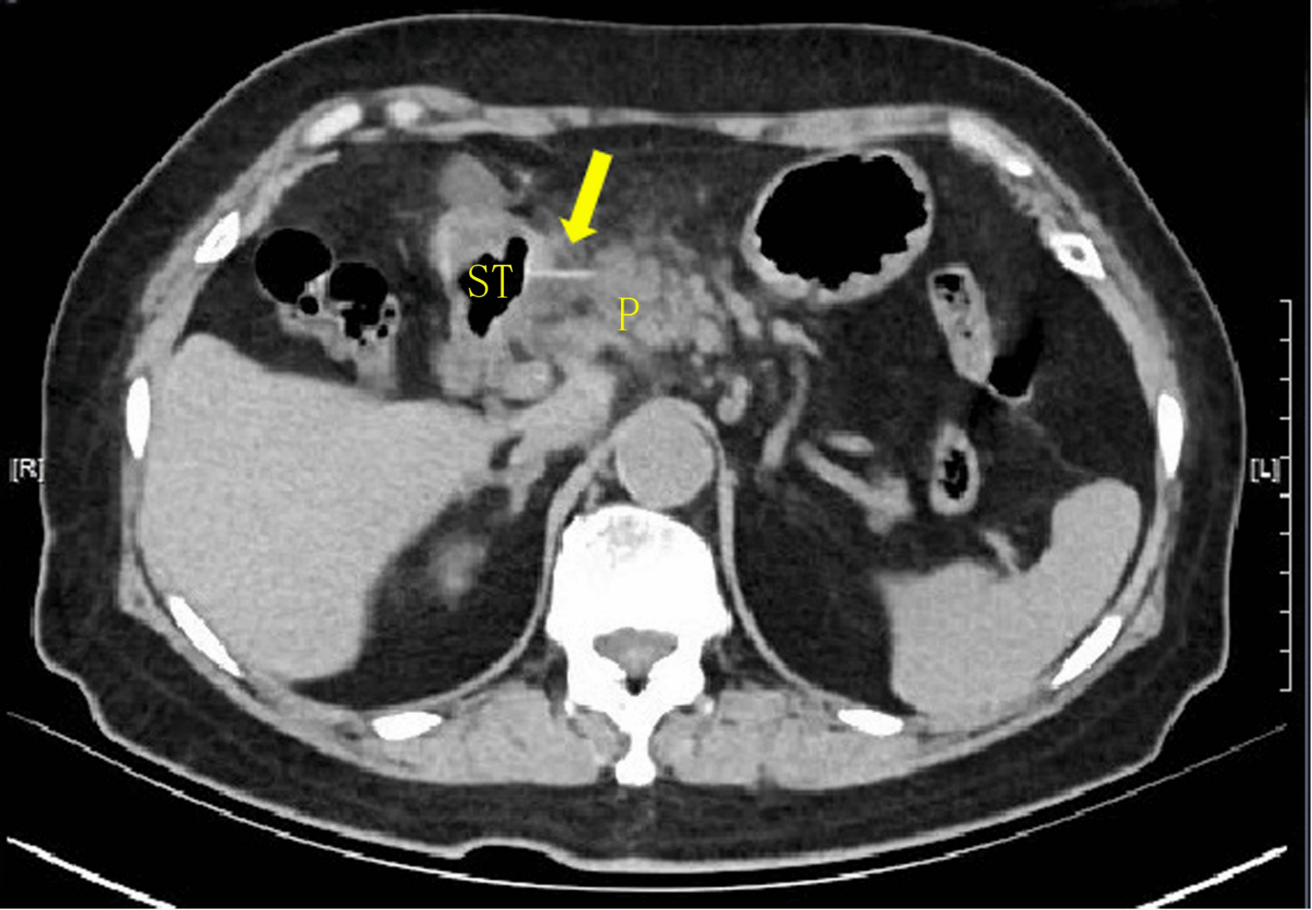
Upper abdominal CT image on admission showing the fishbone (arrow) penetrating the gastric antrum and piercing the head of the pancreas P: pancreas, ST: stomach, CT: computed tomography

**Figure 2:**
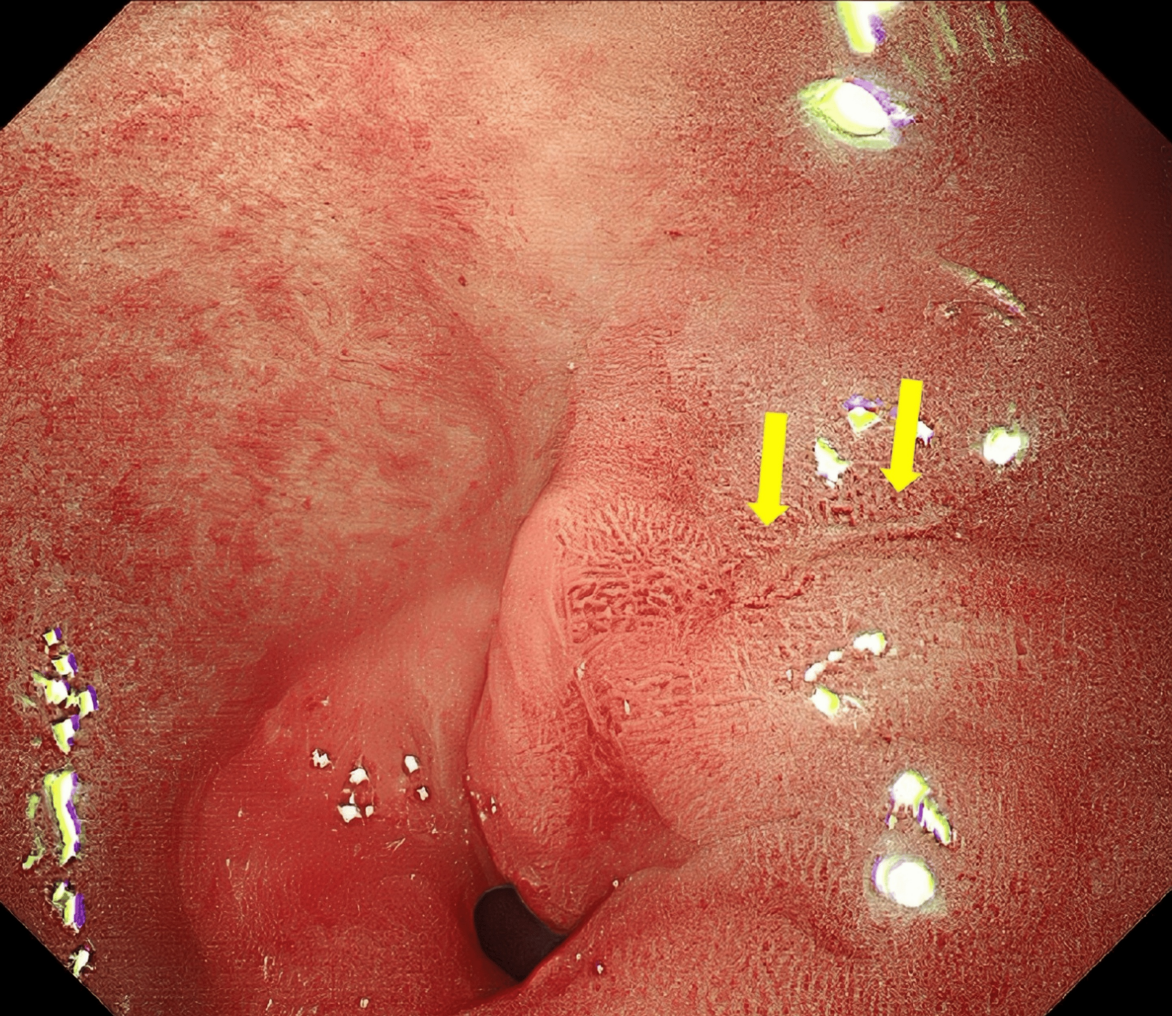
Gastroscopy showing a linear ulcer (arrow) with a length of approximately 6 mm on the posterior wall of the lesser curvature of the gastric antrum

A 73-year-old woman presented to the emergency department with seven days of progressive, persistent epigastric pain without fever. Physical examination revealed mild epigastric tenderness to palpation without rebound tenderness or muscle guarding. Laboratory tests showed mild leukocytosis, elevated C-reactive protein, and normal serum amylase and lipase levels (Table 1). To facilitate the differential diagnosis of conditions such as pancreatitis, cholecystitis, cholangitis, and peptic ulcer disease, a CT scan of the upper abdomen was performed, and it revealed AP, linear high-density shadows suggesting fishbone penetration through the posterior gastric antrum into the pancreatic neck, but no free air or abscess (Figure 1). Clinical and imaging findings supported a diagnosis of mild AP [2,3]. Subsequent gastroscopy identified a linear ulcer (~6 mm) on the posterior wall of the gastric antrum, consistent with foreign body perforation (likely fishbone) with local mucosal damage and edema (Figure 2).

**Table 1:** Laboratory test results

Project name	Test results	Reference value	Unit
Hemoglobin (HGB)	129	115-150	g/L
Red blood cell count (RBC)	4.09	3.8-5.1	10^12^/L
White blood cell count (WBC)	11.33 ↑	3.5-9.5	10^9^/L
Hematocrit (HCT)	38.0	35-45	%
Mean corpuscular volume (MCV)	92.9	82-100	fl
Mean corpuscular hemoglobin (MCH)	31.5	27-34	pg
Mean hemoglobin content (MCHC)	339	316-354	g/L
Platelet count (PLT)	235	125-350	10^9^/L
Platelet hematocrit (X-PCT)	0.21	0.11-0.28	%
Large platelet ratio (P-LCR)	17.0	13-43	%
Red blood cell distribution width CV (RDW-CV)	12.7	11.6-14	%
Red blood cell distribution width SD (RDW-SD)	43.1	39-46	fl
Platelet distribution width (PDW)	10.5	15.1-18.1	fl
Mean platelet volume (MPV)	8.7	9.4-12.5	fl
Neutrophil % (NEUT%)	66.7	40-75	%
Lymphocyte % (LYMPH%)	23.0	20-50	%
Mononuclear cell % (MONO%)	6.3	3-10	%
Eosinophils % (EO%)	3.40	0.4-8	%
Basophil count (BASO#)	0.60	0-1	10^9^/L
neutrophil count (NEU#)	7.56 ↑	1.8-6.3	10^9^/L
Lymphocyte count (LYM#)	2.61	1.1-3.2	10^9^/L
Eosinophil count (EO#)	0.38	1.1-3.2	10^9^/L
Mononuclear cell count (MONO#)	0.71	0.02-0.52	10^9^/L
Basophil count (BASO#)	0.07	0-0.06	10^9^/L
C-reactive protein (CRP)	50.90 ↑	0-8	mg/L
Amylase	82	30-110	U/L
Lipase	52	23-300	U/L

Further history revealed the patient consumed crucian carp one week prior without initial discomfort. Persistent, severe knife-cut pain in the upper abdomen developed two days later. Following the discussion, the patient was transferred to the Department of Gastroenterology, the Second Affiliated Hospital of Naval Medical University, for further care. Alongside continued fluid resuscitation, acid suppression, and antibiotic therapy, endoscopic ultrasound (EUS)-guided retrieval was planned pending reassessment.

**Figure 3:**
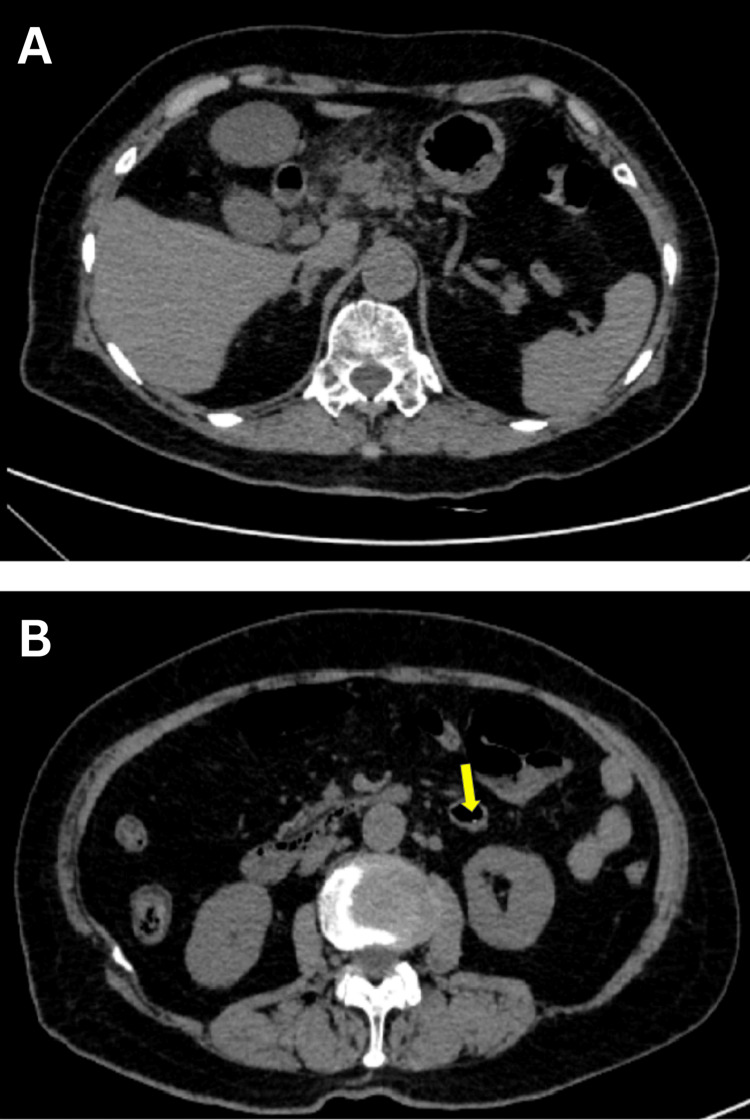
Repeated CT image 48 hours later showing the fishbone no longer in the pancreas, having migrated into the intestinal lumen (A) Upper abdominal CT image showing no fishbone in the gastric antrum and pancreas. (B) Abdominopelvic CT image showing the fishbone in the intestinal lumen. CT: computed tomography

**Figure 4:**
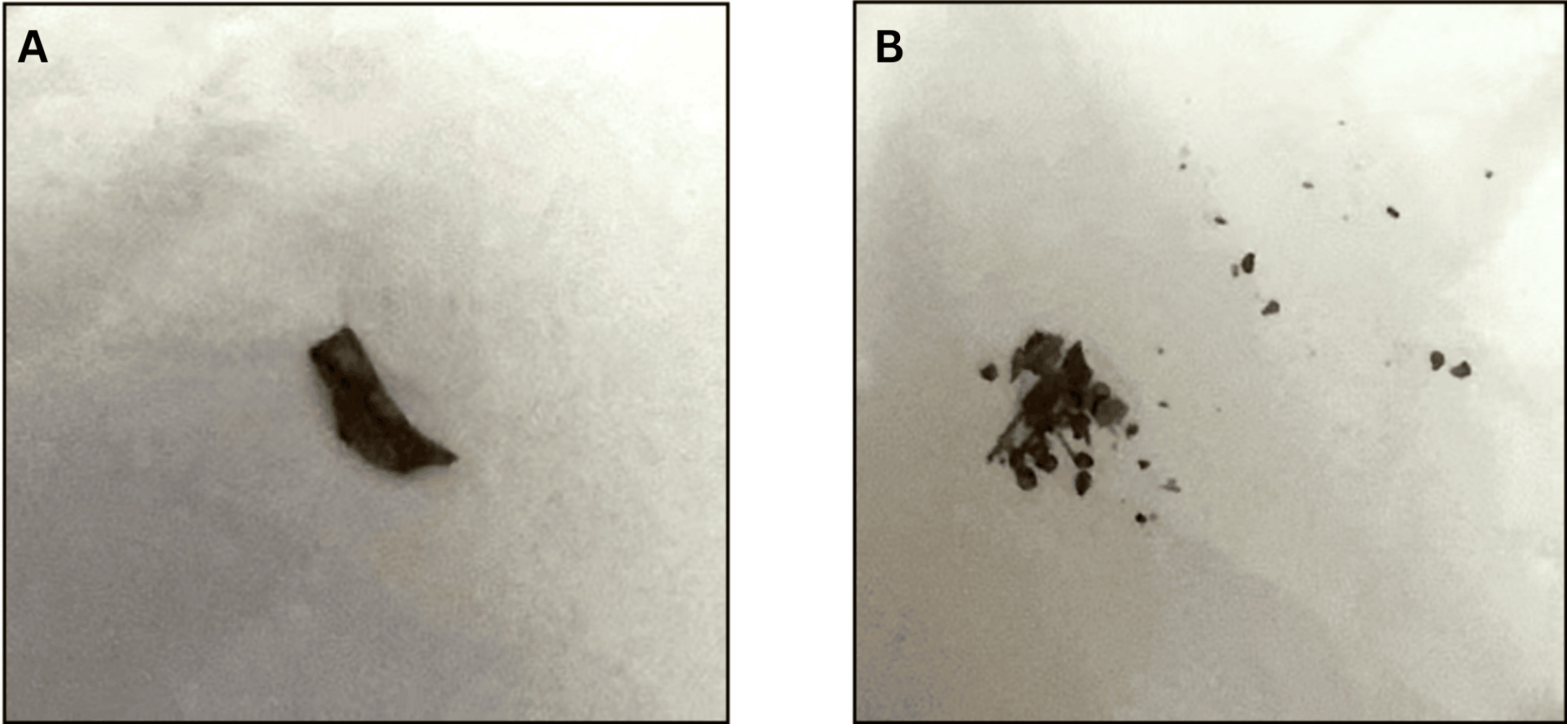
Part of the fishbone expelled through defecation (A) A piece of the fishbone. (B) The crushed fishbone.

Unexpectedly, a repeat upper abdominal CT one day post-transfer showed resolution of the previously observed fishbone shadow (Figure 3A), correlating with the patient's reported pain improvement. To localize the foreign body, an abdominopelvic CT scan was performed the same day, identifying the fishbone within the intestinal lumen (Figure 3B). Given this spontaneous migration and absence of acute complications, conservative management was pursued, and the patient was discharged for observation. On the second post-discharge day, the patient excreted stool and found a fishbone after filtration (Figure 4A), which fragmented during handling (Figure 4B). With the causative agent removed, symptoms resolved rapidly, and oral intake resumed. Follow-up confirmed sustained recovery.

## Discussion

While gallstones, alcohol, hypertriglyceridemia, medications, trauma, and tumors are predominant AP causes [2], foreign body penetration is rare [5]. This case illustrates AP secondary to a fishbone migrating from the stomach into the pancreatic parenchyma, a phenomenon scarcely documented. Foreign body ingestion is common but often unrecognized [6]. Most small objects pass spontaneously within a week [6,10]. Perforation occurs in <1% of cases, with fish/chicken bones implicated in ~50% [8,11]; typical sites are the terminal ileum, sigmoid colon, and rectum [12,13]. Migration into the pancreas is exceptional.

Foreign body-induced AP poses diagnostic challenges due to nonspecific symptoms (upper abdominal pain, nausea, vomiting) mimicking common etiologies. In this case, symptom onset delay and normal pancreatic enzymes (potentially due to prolonged duration and localized inflammation) further complicate diagnosis. CT is essential, demonstrating the penetrating foreign body and inflammation. Enhanced CT remains the gold standard for radiopaque objects and complication assessment. Radiolucent objects may require EUS or MRI. Crucially, clinicians must correlate imaging with dietary history, as patients often overlook ingestion events.

Nearly all documented cases of pancreatitis caused by fishbone impaction required surgical management [8,9]. In this case, the fishbone spontaneously migrated into the bowel lumen, likely facilitated by intestinal peristalsis, and prevented invasive procedures. Generally, management hinges on timely foreign body removal, infection control, and pancreatic rest. Intervention depends on foreign body location and complications. Delayed intervention risks severe complications (necrotizing pancreatitis, sepsis, fistulae). Endoscopy is first-line for accessible intraluminal objects [13]. However, objects fully embedded in pancreatic parenchyma or severe sequelae like abscesses necessitate surgical intervention [8,9], typically involving laparotomy, foreign body extraction, perforation repair, and debridement. Postoperative care includes fluid resuscitation, broad-spectrum antibiotics, and enteral nutrition. Lately, with the development of new techniques, it is possible to attempt to treat extraluminal foreign bodies using minimally invasive techniques, such as EUS. As is reported, the location of ingested objects traversing the gastrointestinal wall can be accurately determined by EUS, which may provide a therapeutic alternative to conventional surgery with lower associated risks of complications and mortality [14]. In this case, the initial plan for EUS-guided retrieval of the foreign body was not implemented, as it spontaneously migrated into the intestinal lumen, requiring no further intervention. Nevertheless, standardized protocols and comparative studies on endoscopic versus surgical approaches are needed.

## Conclusions

This case demonstrates a rare case of AP caused by a fishbone penetrating the gastric antrum into the pancreas. Spontaneous migration into the intestinal lumen facilitated natural expulsion, averting invasive intervention. This case underscores the critical roles of early imaging, rapid diagnosis, inter-hospital or multidisciplinary cooperation, and prompt appropriate treatment, which are pivotal in avoiding fatal complications. For embedded pancreatic foreign bodies, endoscopic or surgical treatment could be an effective option. Pre-procedural re-evaluation is essential to avoid unnecessary interventions if spontaneous migration occurs.
